# MASM, a Matrine Derivative, Offers Radioprotection by Modulating Lethal Total-Body Irradiation-Induced Multiple Signaling Pathways in Wistar Rats

**DOI:** 10.3390/molecules21050649

**Published:** 2016-05-17

**Authors:** Jianzhong Li, Jing Xu, Yiming Lu, Lei Qiu, Weiheng Xu, Bin Lu, Zhenlin Hu, Zhiyong Chu, Yifeng Chai, Junping Zhang

**Affiliations:** 1 School of Pharmacy, Second Military Medical University, Shanghai 200433, China; xujing0601103@live.cn (J.X.); bluesluyi@163.com (Y.L.); qlcong021@163.com (L.Q.); xuweiheng7114@163.com (W.X.); binlu@smmu.edu.cn (B.L.); zhenlinhu@hotmail.com (Z.H.); yfchai@smmu.edu.cn (Y.C.); 2 Department of Pharmacy, East Hospital, Dongji University, Shanghai 200085, China; 3 The Naval Medical Research Institute, Shanghai 200433, China; zhiyongchu@tom.com

**Keywords:** MASM, radiation lethality, total-body irradiation, radioprotection, gene expression, MAPK pathways

## Abstract

Matrine is an alkaloid extracted from *Sophora flavescens* Ait and has many biological activities, such as anti-inflammatory, antitumor, anti-fibrosis, and immunosuppressive properties. In our previous studies, the matrine derivative MASM was synthesized and exhibited potent inhibitory activity against liver fibrosis. In this study, we mainly investigated its protection against lethal total-body irradiation (TBI) in rats. Administration of MASM reduced the radiation sickness characteristics and increased the 30-day survival of rats before or after lethal TBI. Ultrastructural observation illustrated that pretreatment of rats with MASM significantly attenuated the TBI-induced morphological changes in the different organs of irradiated rats. Gene expression profiles revealed that pretreatment with MASM had a dramatic effect on gene expression changes caused by TBI. Pretreatment with MASM prevented differential expression of 53% (765 genes) of 1445 differentially expressed genes induced by TBI. Pathway enrichment analysis indicated that these genes were mainly involved in a total of 21 pathways, such as metabolic pathways, pathways in cancer, and mitogen-activated protein kinase (MAPK) pathways. Our data indicated that pretreatment of rats with MASM modulated these pathways induced by TBI, suggesting that the pretreatment with MASM might provide the protective effects on lethal TBI mainly or partially through the modulation of these pathways, such as multiple MAPK pathways. Therefore, MASM has the potential to be used as an effective therapeutic or radioprotective agent to minimize irradiation damages and in combination with radiotherapy to improve the efficacy of cancer therapy.

## 1. Introduction

Radiation source is a kind of physical stress that humans risk in nuclear pollution, space flight and radiation therapy for cancer events [1,2,3]. Radiation exposure increases the oxidative pressure and induces further damages such as DNA lesions, cell death, cancer and other diseases. Radioprotective agents are administered either before or after radiation exposure to minimize radiation toxicity. Several compounds have been reported to offer potential for radiation protection, but most of them are not suitable for clinical application due to their toxicity and poor specificity [4,5,6]. This warrants development of suitable radioprotective agents with minimum toxicity that can be used under occupational as well as clinical conditions.

Matrine (molecular formula: C_15_H_24_N_2_O) is an alkaloid found in the herb root of a traditional Chinese medicine, *Sophora flavescens* Ait [7,8]. It has a variety of pharmacological effects and has been wildly used in clinical practice in China for the treatment of viral hepatitis, liver fibrosis, heart arrhythmia and skin inflammation without any obvious side effects [7,8,9,10,11,12,13]. In addition, many studies have shown that matrine possesses strong antitumor activities *in vitro* and *in vivo* [8,14]. Matrine and its compounds also have been extensively used alone or in combination with chemotherapy or radiotherapy for many years in China [14]. Clinical studies have shown that matrine and its compounds attenuate the side effects of chemotherapy and radiotherapy by improving the life quality and regulating the immunologic function of patients with cancer, as well as synergizing the therapeutic effects of chemotherapy and radiotherapy [14,15,16,17,18]. In our previous studies, the matrine derivative MASM ((6a*S*,10*S*,11a*R*,11b*R*,11c*S*)-10-methylamino-dodecahydro-3a,7a-diazabenzo(de)anthracene-8-thione) was synthesized via classical Michael addition and exhibited potent antiinflammatory and inhibitory activity against liver fibrosis *in vitro* and *in vivo* associated with the reduction of Akt phosphorylation [19,20]. In this study, we mainly investigate its protection against lethal TBI in Wistar rats.

## 2. Results and Discussion

### 2.1. Effect of MASM on 30 Day Survival

**Figure 1:**
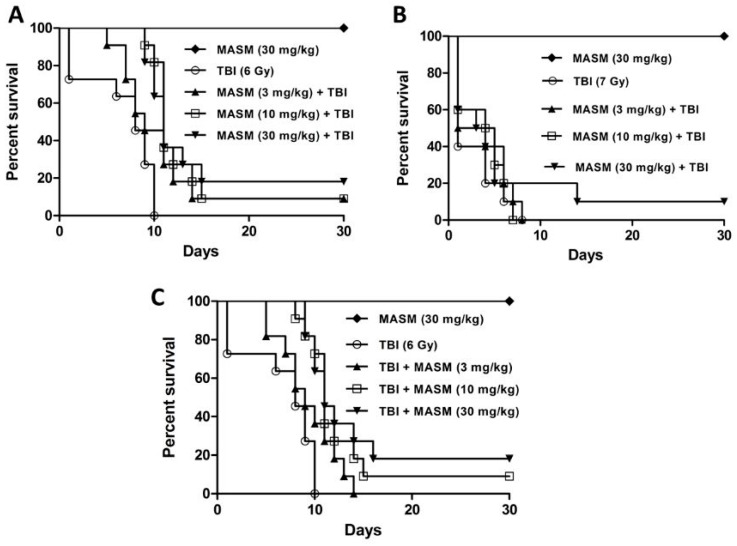
Comparison of survival curves by using Kaplan-Meier method. (**A**) 30 days survival of rats (*n* = 11 per group) pretreated with MASM (3 mg/kg, 10 mg/kg and 30 mg/kg) once each day for three consecutive days before exposure to 6 Gy TBI. The survival curves were significantly different as predicted by log rank test (*p* = 0.0011); (**B**) 30 d survival of rats (*n* = 11 per group) pretreated with MASM (3mg/kg, 10 mg/kg and 30 mg/kg) once each day for three consecutive days before exposure to 7 Gy TBI; (**C**) 30 days survival of rats (*n* = 11 per group) treated with MASM (3 mg/kg, 10 mg/kg and 30 mg/kg) once each day for three consecutive days 30 min after exposure to 6 Gy TBI. The survival curves were significantly different as predicted by log rank test (*p* = 0.0015).

The radiation control group exhibited typical signs of radiation sickness such as reduced intake of food and water, irritability, lethargy, weight loss, ruffling of hair, diarrhea, emaciation and epilation with median survival of only 8 days and 100% mortality within 10 days (Figure 1A). MASM (30 mg/kg) alone did not induce any noticeable sign of toxicity within 30 days. On the other hand, rats that were administered with different graded concentrations of MASM once a day for three consecutive days prior to the exposure of lethal TBI (6 Gy) had reduced signs of radiation sickness and improved survival rates. Different concentrations of MASM were found to offer same protection to rats against radiation induced toxic effects by increasing the median survival period to 11 days. Pre-administration of 3, 10 and 30 mg/kg MASM reduced the radiation sickness characteristics, while increasing the 30-day survival of the irradiated rats by 9%, 9% and 18% respectively (Figure 1A). The log rank test indicated that the survival curves were significantly different (** *p* < 0.01). However, when rats were exposed to a lethal dose of 7 Gy TBI, 3 mg/kg and 10 mg/kg MASM failed to show increase 30-day survival (Figure 1B). In additional, the log rank test indicated that the survival curves were non-significantly different.

Encouraged by the above results, the therapeutic ability of MASM was also evaluated by administering it 30 min after exposure to a lethal dose (6 Gy) of TBI. Rats that were administered with different concentrations of MASM once each day for three consecutive days after TBI had reduced signs of radiation sickness and increased median survival (Figure 1C). Administration of MASM 10 mg/kg and 30 mg/kg increased the 30 day-survival of the irradiated mice by 9% and 18% respectively, confirming its therapeutic ability (Figure 1C). The log rank test indicated that the survival curves were significantly different (** *p* < 0.01).

### 2.2. Effect of MASM on Radiation-Induced Tissues Injury Using Transmission Electron Microscopy

To observe the possible protective effect of MASM on radiation-induced tissues injury in rats, transmission electron microscopy was performed. Rats (*n* = 10 rats/group) received 30 mg/kg/day MASM or water once each day for three days prior to radiation. Significant pathological changes occurred at 1 day after lethal TBI (6 Gy).

**Figure 2:**
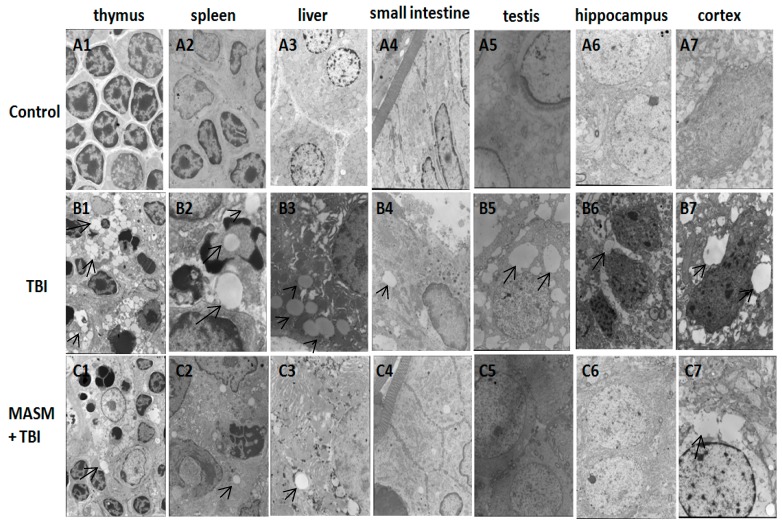
Effect of MASM on radiation-induced tissues injury at 1 day after exposure to lethal TBI (6 Gy) using transmission electron microscopy. (**A1**–**7**) Representative photographs of non-irradiated rats; (**B1**–**7**) Representative photographs of 10 TBI rats. Cell swelling and large amounts of cytoplasmic vacuoles were observed in the different tissues at 1 day after exposure to lethal TBI (6 Gy); (**C1**–**7**) Representative photographs of 10 irradiated rats pretreated with MASM (30 mg/kg) once each day for three consecutive days before exposure to 6 Gy TBI. MASM pretreatment attenuated the radiation-induced tissues injury such as cytoplasmic vacuoles (arrows). (uranyl acetate and lead citrate staining, 3000×).

Damages in thymus, spleen, liver, small intestine, testis, hippocampus and cortex such as cytoplasmic vacuolization, dilatation of the endoplasmic reticulum and destruction of mitochondria, as well as damages to the cellular membrane, were observed (Figure 2B1–7). In addition, the morphological signs of apoptosis were frequently detected in the splenocytes. Apoptotic splenocytes showed the marginal condensation of chromatin onto the nuclear lamina (Figure 2B2). Vacuoles and mitochondrial swelling were decreased by MASM in the different organs (Figure 2C1–7). Cell apoptosis also significantly attenuated by MASM in the spleen (Figure 2C2). These results illustrated that pretreatment of rats with MASM before exposure to acute lethal TBI significantly attenuated the radiation-induced morphological changes in the irradiated different organs, confirming the protective effects of MASM against radiation-induced tissue injury.

### 2.3. MASM Prevents TBI-Induced Differential Expression of Many Genes

The liver was considered to be a radiosensitive organ [21]. Vacuoles and mitochondrial swelling were significantly decreased by MASM in the liver (Figure 2C3), indicating that MASM ameliorated TBI-induced liver tissues injury. To further investigate the potential molecular basis of the protective effects of MASM on irradiation damage, gene expression analysis was conducted on rats liver tissues using microarrays.

**Figure 3:**
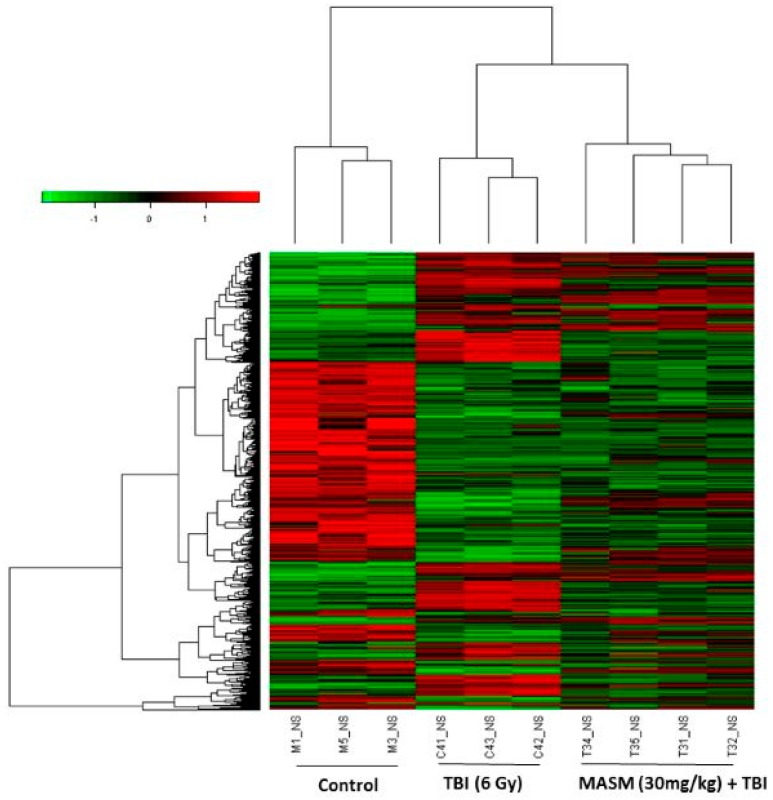
Cluster analysis of individual rats according to the profile of gene expression examined. Genes are organized by hierarchical clustering based on overall similarity in expression patterns. Red represents relative expression greater than the median expression level across all samples, and green represents an expression level lower than the median. Black indicates intermediate expression. Rats from the same treatment group (6 Gy TBI, 30 mg/kg MASM + 6 Gy TBI) were the closest.

A heatmap was generated (Figure 3) representing 3912 transcripts (probesets) comparing significantly differentially regulated genes (*p* < 0.01) for the treatment groups (TBI, MASM + TBI) *versus* untreated controls by using one-way analysis of variance (one-way ANOVA) (Supplementary File 1). Cluster analysis of the microarray results revealed that three or four rats from the same group were the closest, indicating that all the experimental data in this study were reliable.

A combined algorithm with simple *t*-test and fold change was used to find differentially expressed genes. By a threshold of *p* < 0.05 and absolute fold change (FC) > 1.5, 2920 significant expressed probes were selected by comparison between radiation and wild-type (control) samples, involving 1445 unique genes (Supplementary File 2). Among the genes originally displaying FC > 1.5, 414 were subsequently down-regulated and 1031 up-regulated. In the pretreatment with 30 mg/kg MASM groups, only 680 out of 1445 genes were still differentially expressed in response to TBI. These results indicated that the treatment with MASM prevented TBI-induced differential expression of 53% (765 genes) of genes (Supplementary File 3). We focused on these genes for which TBI-induced alteration of expression were abolished or attenuated by MASM pretreatment, as these genes might be regulated by MASM and involved in the protective effects on radiation.

Gene ontology (GO) analysis was then applied to these genes in terms of molecular function, cellular component and biological process. Eleven enriched terms associated with these genes list were identified (Table 1). The identified terms in molecular function were transporter activity, and chemorepellent activity. Only synapse part was in cell component. The terms included in biology process were death, growth, multicellular organismal process, locomotion, rhythmic process, response to stimulus, and localization. 

Pathway enrichment analysis indicated that these genes were mainly involved in a total of 21 pathways (Table 2). Most of these pathways were reported to be induced by radiation in the previous studies, such as olfactory transduction [22], cytokine-cytokine receptor interaction [22], neuroactive ligand-receptor interaction [23], pathways in cancer [24,25,26], MAPK signaling pathway [27], PPAR signaling pathway [28], GnRH signaling pathway [29], calcium signaling pathway [25], acute myeloid leukemia [30,31], vascular smooth muscle contraction [32], gap junction [33], bladder cancer [34,35] and circadian rhythm [36,37]. Interestingly, recently a review article reported that targeting cellular metabolism can improve the efficacy of cancer therapy [38].

For example, targeting metabolic enzymes, such as glucose transporters, fatty acid synthase and glutaminase can enhance the efficacy of common therapeutic agents or overcome resistance to chemotherapy or radiotherapy [38]. In addition, it has been reported that nicotinamide sensitizes tumors, at least in part, by modulating vascular smooth muscle contraction [39]. Matrine and its compounds have been widely used as adjuvant therapy in China to improve the 5-year survival rate and life quality of patients with cancer [14]. However, little is known about the mechanisms underlying the therapeutic effects of matrine. Table 2 showed that 38, 10, 7, 5 and 12 genes were involved in metabolic pathways, purine metabolism, arachidonic acid metabolism, fatty acid metabolism and vascular smooth muscle contraction, respectively. It is very likely that MASM/matrine improve the efficacy of cancer therapy, at least in part, by targeting these cellular metabolism and modulating vascular smooth muscle contraction, and this area is of great interest for our further studies. 

Since ionizing radiation induces simultaneous compensatory activation of multiple MAPK pathways [27], we focused on these pathways. These pathways play critical roles in controlling cell survival or death and repopulation effects following irradiation, in a cell-type-dependent manner [27]. The 13 genes presented in Table 3 were involved in multiple MAPK pathways including extracellular signal-regulated kinase (ERK), c-Jun N-terminal kinase (JNK), and p38 MAPK pathway. 

This was consistent with previous reports that ionizing radiation activated all 3 MAPKs with different intensities and in a cell type-dependent context [27,40,41]. 11 of among gene expressions were evidently up-regulated by TBI and these up-regulations were abolished or attenuated by pretreatment with MASM. These TBI-induced genes include fibroblast growth factor 3 (Fgf3), myelocytomatosis oncogene (Myc), phospholipase A2, group IIA (Pla2g2a or sPLA2), growth arrest and DNA-damage-inducible, beta (Gadd45b), mitogen activated protein kinase kinase kinase 10 (Map3k10), protein kinase, X-linked (Prkx), calcium channel, voltage-dependent, R type, alpha 1E subunit (Cacna1e), calcium channel, voltage-dependent, L type, alpha 1S subunit (Cacna1s), TAO kinase 2 (Taok2), activating transcription factor 4 (Atf4), nuclear receptor subfamily 4, group A, member 1 (Nr4a1). In contrast, the expressions of neurotrophic tyrosine kinase, receptor, type 1 (Ntrk1) and apoptosis signal-regulating kinase 1 (Ask1) were down-regulated by TBI and these down-regulations were abolished or attenuated by pretreatment with MASM. Interestingly, Nr4a1 was a growth factor-inducible member of the Nr4a1/Nur77 subfamily of the nuclear receptor superfamily of transcription factors. Nr4a1 was involved in determine whether cells undergo double-strand break (DSB) repair or apoptosis in response to irradiation [42]. A previous study reported that radiation up-regulated Nr4a1/Nur77 phosphorylation and expression [42]. In this study, our data indicated that Nr4a1 expression was evidently up-regulated by TBI and the TBI-induced increase was attenuated by pretreatment with MASM. In addition, activating transcription factor 4 (Atf4) was a member of the ATF/CREB (activating transcription factor/cyclic AMP response element binding protein) family of basic region-leucine zipper (bZip) transcription factors. Atf4 was induced by stress signals including endoplasmic reticulum stress, amino acid deprivation, anoxia/hypoxia and oxidative stress. Atf4 expression was regulated transcriptionally, translationally via the PERK pathway. Atf4 regulated the expression of genes involved in amino acid synthesis, oxidative stress, differentiation, metastasis and angiogenesis [43]. As described by the earlier reports [44,45], our data indicated that TBI increased the expression of Atf4/CREB. Myc was involved in a wide range of cellular processes including signal transduction, cell-cycle control, self-renewal, metabolism, maintenance of pluripotency, and control of cell fate decisions [46]. Here, our data indicated that the TBI-induced increase in Myc was attenuated by pretreatment with MASM. This was consistent with previous reports that irradiation significantly increased Myc [47,48]. Gadd45 proteins were implicated in stress signaling in response to environmental or physiological stressors, which resulted in either cell cycle arrest, DNA repair, cell survival and senescence, or apoptosis [49]. Consistent with the earlier reports [50,51], the expression of Gadd45b was up-regulated by radiation. Prkx was a member of an ancient family of cAMP-dependent serine/threonine kinases distinct from the classical PKA, PKB/Akt, PKC, SGK, and PKG families [52]. As described by the earlier reports [53,54], Prkx protein kinase was up-regulated by radiation. Ntrk1/Trk A was a member of the neurotrophic tyrosine kinase receptor (NTKR) family. Ntrk1 was a membrane-bound receptor that, upon neurotrophin binding, phosphorylates itself and members of the MAPK pathway. The presence of Ntrk1 leaded to cell differentiation and might play a role in specifying sensory neuron subtypes [55,56]. In UV-irradiated normal skin, there was a significant reduction in Trk A/Ntrk1 tyrosine kinase receptor immunostaining after UV-irradiation [57]. Albeit our data strongly support radiation-induced down-regulation of Ntrk1 [57], there are also reports on UV-induced up-regulation of both nerve growth factor NGF and its high-affinity receptor Ntrk1 [58]. cPLA2 was a member of the PLA2 enzyme superfamily, which included secretory PLA2 (sPLA2), cytosolic PLA2 (cPLA2), and other members. cPLA2, which activated AA hydrolysis, existed in three isoforms: α, β, and γ. cPLA2-α was known to be a major component of the arachidonate-releasing signal transduction pathway [59,60]. Low level laser irradiation significantly inhibited phospholipase cPLA2-α mRNA expression, which was increased in response to mechanical stress [59]. Here, our data showed that TBI-induced increase in sPLA2 was attenuated by pretreatment with MASM. These data showed that MASM pretreatment could attenuate TBI-activated all 3 MAPKs (Table 3). In our previous study, MASM could inhibit the Akt pathway by suppressing the phosphorylation of Akt, glycogen synthase kinase 3β (GSK3β), and P70S6 kinase in cultured activated or TGFβ1-activated hepatic stellate cells [20]. In addition, our data showed that radiation-induced phosphorylation of P38 and JNK (c-Jun NH_2_-terminal kinase) was dose-dependently inhibited by pretreatment with MASM in murine macrophage RAW264.7 cells (unpublished data). Moreover, some agents were reported to provide the protective effects via the modulation of MAPKs pathway [61,62,63,64]. Taken together, these data indicated that the pretreatment of rats with MASM modulated lethal TBI-induced multiple MAPK pathways, suggesting that MASM might provide the protective effects mainly or partially through the modulation of MAPKs pathway. Therefore, MASM has the potential to be used as an effective therapeutic or radioprotective agent to minimize TBI-induced damages.

A number of studies demonstrated that the combined modality therapy involving radiation and MAPK pathway inhibitors was a promising strategy for improving the treatment of patients with cancer [40]. Preclinical and clinical evidence suggested that agents targeting aberrant tumor signals could effectively improve the therapeutic effect of ionizing radiation [24]. Here, this study suggested that MASM could offer radioprotection mainly or partially through the modulation of TBI-induced MAPKs pathway. Therefore, it is very likely that MASM is used in combination with radiotherapy to improve the efficacy of cancer therapy, and this area is of great interest for our further studies.

**Figure 4:**
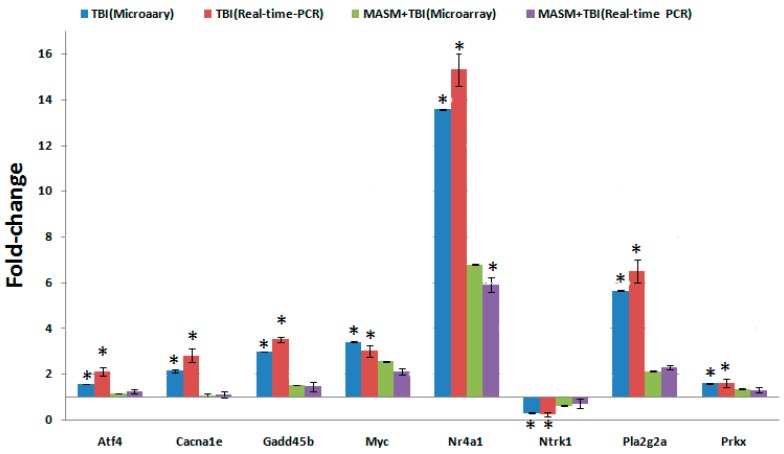
Quantitative real-time PCR confirmation of the microarray data. qRT-PCR was performed on 8 genes that TBI-induced alteration of expression was abolished or attenuated by 30 mg/kg MASM pretreatment. RNA samples of different groups (*n* = 5 per group) were prepared 24 h after exposure to 6 Gy TBI. Gene expression levels are shown as the mean normalized to the expression of the housekeeping gene beta-actin. Each sample was measured in triplicate. Columns, mean of three or four rats in the microarray experiment or mean of five rats in PCR; bars, SD. * indicates statistical significance compared to control (non-irradiation) by *t*-test, * *p* < 0.05. Comparison of fold change produced by microarray with relative expression ratio obtained from real-time PCR, with good concordance.

To validate the consistency of microarray analysis in the present study, we compared gene expression levels of selected genes between microarray and real-time PCR. We determined the mean value of expression of the selected genes in five independent rats from each exposure group. This was compared with those in pooled RNA from 5 non-irradiated rats. The qualitative changes in gene expression levels were consistent between these analyses (Figure 4). 

## 3. Materials and Methods

### 3.1. Chemicals

The matrine derivative MASM [(6a*S*,10*S*,11a*R*,11b*R*,11c*S*)-10-methylamino-dodecahydro-3a,7a-diazabenzo(de)anthracene-8-thione] (purity > 99%) was synthesized via classical Michael addition and characterized as reported earlier [19].

### 3.2. Animals 

Male Wistar rats (200–220 g) were purchased from SLAC Laboratory Animal Co. Ltd. (Shanghai, China) and divided randomly into several groups. Animals were kept under standard laboratory conditions of temperature, pressure and humidity. Food and water were sterilized by ^60^Co γ-irradiation and high pressure, respectively. All animal procedures were carried out in strict accordance with the recommendations in the Guide for the Care and Use of Laboratory Animals of the National Institutes of Health. The protocol was approved by the Committee on the Ethics of Animal Experiments of the Second Military Medical University (Shanghai, China). All surgery was performed under sodium pentobarbital anesthesia, and all efforts were made to minimize suffering.

### 3.3. Radiation and Administration

The animals were randomly assigned to one of the six following treatment groups (10–11 animals per group): normal control, MASM-High, radiation and MASM-High, Medium, Low dose (30, 10, 3 mg/kg body weight/day) + radiation. Different doses of MASM dissolved in double distilled water were administered intragastrically to the animals once each day for 3 consecutive days before or after irradiation. All animals, except the normal control group, were placed in specially designed, well-ventilated acrylic container and subjected to total-body irradiation (TBI). Radiation was delivered by the ^60^Co source (Radiation facility, the Second Military Medical University, China). Radiation doses were 6, 7 Gy at a rate 2 Gy/min. The animals were monitored daily for the development of symptoms of radiation sickness and mortality.

### 3.4. Survival Assays 

Wistar rats (*n* = 11 rats /group) were administered MASM after or before exposure to TBI with 6 Gy or 7 Gy. We observed animals twice times daily for a period of 30 days to determine survival rates, and moribund animals were euthanized according to humane endpoints [65]. The clinical criteria of moribund is being in the state of dying with no expectation of recovery, where animals display a combination of the following: hunched back, lowered body temperature, impaired or slow motion, continuous shaking and inability to maintain sternal recumbency [65,66]. Moribund and surviving animals at the end of the study were subjected to euthanasia by the application of sodium pentobarbital anesthesia or CO_2_ followed by cervical dislocation. Survival of rats or mice from all the different groups was monitored from the day of onset of the experiment until the 30th day. The probabilities of the survival of all different groups were plotted as Kaplan-Meier survival curves until the 30th day. Significant differences in the survival curves among the different groups were evaluated using log rank test (Mantel-Cox test) for multiple groups.

### 3.5. Sample Collection

24 h following irradiation, the rats were anesthetized and sacrificed by cervical dislocation. Thymus, spleen, liver, small intestine, testis, hippocampus, cortex and femoral were dissected out from each animal. The specimens of all groups were processed for ultrastructural examination. In addition, liver tissues of all groups were processed for the microarray experiment.

### 3.6. Transmission Electron Microscopy 

Transmission electron microscopy was performed essentially as described previously [67]. Biopsy samples were cut and fixed in 2.5% glutaraldehyde in 0.1 M sodium cacodylate, then post fixed with 1% osmium tetroxide. The specimens were dehydrated through a graded series of ethanol and then embedded in labeled capsules with freshly prepared resin and left to polymerize. Ultrathin sections were cut on an Ultracut UCT (Leica, Wetzlar, Germany) stained with uranyl acetate and lead citrate and examined by a 1200EX transmission electron microscope (JEOL, Peabody, MA, USA).

### 3.7. Gene Expression Microarray and Data Analysis

The microarray experiments were performed as described previously [68]. The 4 × 44 K Whole Rat Genome Oligo Microarray (Agilent Technologies, Santa Clara, CA, USA) was hybridized with Cy3-labeled cRNA using a Gene Expression Hybridization Kit (Agilent Technologies) in a Hybridization Oven (Agilent Technologies), according to the manufacturer’s instructions. Raw data were obtained by Feature Extraction software 10.7 (Agilent Technologies) and normalized by Quantile algorithm, Gene Spring Software 11.0 (Agilent Technologies). The microarray experiments were conducted at the National Engineering Center for Biochips in Shanghai, China, according to the procedures in the Agilent technical manual. After normalization, genes in the treatment groups with at least 1.5-fold change in expression were considered as up-regulated or down-regulated in comparison to non-treated groups (control). To determine significant proportions of differentially expressed genes within treated groups, the hypergeometric probability *p* was calculated. *p* < 0.05 was considered significant.

Microarray data analysis was performed using the SBC Analysis system, which is available on the website: http://www.ebioservice.com/. The username and password to access this website are available upon request. A general description of the SBC analysis system can be found on the website: http://www.ebioservice.com/sas.html. The microarray data generated in this study have been deposited in the Gene Expression Omnibus (GEO) database under the accession number GSE56263.

### 3.8. Quantitative Real Time (qRT)-PCR Array Validation 

qRT-PCR was performed essentially as described previously[68]. In total, eight genes were chosen for RT-PCR validation. Because SYBR Green binding is not sequence specific, careful design and validation of each primer pair, as well as cautious manipulation of RNA were undertaken to ensure that only target gene sequence-specific, non-genomic products were amplified by real-time PCR. To achieve this, primers were designed to either span or flank introns by using the ProbeFinder version 2.49 software (Roche, West Sussex, UK). A dissociation curve analysis was performed at the end of the amplification process in order to verify the specificity of the PCR products. The same PCR products were also evaluated by agarose gel electrophoresis. Data are presented as mean ± SD. The primers are as follows. Atf4 (NM_024403): forward, TCAGACACCGGCAAGGAG; reverse, GTGGCCAAAAGCTCATCTG. Cacna1e (NM_019294): forward, GAAATTATCCTGACAGACAGCAAG; reverse, AGCTTTATGAGCCGTGCAG. Gadd45b (NM_001008321): forward, CTGCCTCCTGGTCACGAA; reverse, TTGCCTCTGCTCTCTTCACA. Myc (NM_012603): forward, GAATTTTTGTCTATTTGGGGACA; reverse, GCATCGTCGTGACTGTCG. Nr4a1 (NM_024388): forward, TGTTGATGTTCCTGCCTTTG; reverse, GGAGGCCATGTCGATCAG. Ntrk1 (NM_021589): forward, CAGCTTCTGGCTGTGGCTA; reverse, AAGTGCAGGCTGGCTAGGTA. Pla2g2a (NM_031598): forward, CTGACCTACAAGTTCTCCTACCG; reverse, TTATCGCACTGGCACAGC. Prkx (NM_001033963): forward, GCCTGGGCAACATGAAGA; reverse, TCCACACCTCGGAACCAC. β-Actin (NM_031144): forward, CCCGCGAGTACAACCTTCT; reverse, CGTCATCCATGGCGAACT.

### 3.9. Statistical Analysis

Data were presented as mean ± SD. One-way analysis of variance (ANOVA) followed by the Dunnett’s test was used to examine differences among the different treatment groups. Differences were considered significant when *p* < 0.05 or *p* < 0.01.

## Figures and Tables

**Table 1:** GO analysis of 765 genes which TBI-induced alteration of expression were abolished or attenuated by MASM pretreatment. GO analysis was applied in terms of molecular function, cellular component and biological process (*p* < 0.05).

GOId	Name	Hits	Percent	Enrichment Test *p* Value
GO:0003674	molecular_function	550	3.7%	1.0
GO:0005215	transporter activity	65	6.07%	0.0094
GO:0045499	chemorepellent activity	2	40.0%	0.0331
GO:0005575	cellular_component	555	3.78%	1.0
GO:0044456	synapse part	24	6.72%	0.0376
GO:0008150	biological_process	518	3.76%	1.0
GO:0016265	death	75	5.69%	0.0219
GO:0032501	multicellular organismal process	275	5.1%	0.0054
GO:0032502	developmental process	204	5.02%	0.0324
GO:0040007	growth	36	6.04%	0.0484
GO:0040011	locomotion	54	6.41%	0.0068
GO:0048511	rhythmic process	26	11.02%	0.0001
GO:0050896	response to stimulus	236	4.96%	0.0344
GO:0051179	localization	174	5.12%	0.0281

**Table 2:** Pathway enrichment analysis of 765 genes which TBI-induced alteration of expression were abolished or attenuated by MASM pretreatment. KEGG pathway analysis indicated that these genes were mainly involved in a total of 21 pathways (*p* < 0.01).

Name	Hits	Percent	Enrichment Test *p* Value
Olfactory transduction	58	5.69%	0.0
Metabolic pathways	38	3.15%	0.0091
Neuroactive ligand-receptor interaction	18	5.54%	0.0003
Pathways in cancer	16	4.79%	0.0024
MAPK signaling pathway	13	4.73%	0.0065
Calcium signaling pathway	13	6.81%	0.0003
Vascular smooth muscle contraction	12	9.38%	0.0
Cytokine-cytokine receptor interaction	12	4.88%	0.0070
Purine metabolism	10	5.99%	0.0035
PPAR signaling pathway	9	12.0%	0.0001
GnRH signaling pathway	8	8.08%	0.0016
Gap junction	8	9.2%	0.0007
Arachidonic acid metabolism	7	9.46%	0.0013
Adipocytokine signaling pathway	7	10.45%	0.0008
RNA degradation	6	9.84%	0.0024
Type II diabetes mellitus	5	9.43%	0.0065
Fatty acid metabolism	5	11.11%	0.0034
Acute myeloid leukemia	5	8.47%	0.0098
Galactose metabolism	4	16.67%	0.0024
Circadian rhythm-mammal	4	30.77%	0.0003
Bladder cancer	4	10.81%	0.0095

**Table 3:** The 13 genes were involved in MAPK pathways. These genes that TBI-induced alteration of expression were abolished or attenuated by 30 mg/kg MASM pretreatment.

GenBank	Symbol	Description	Fold Change TBI (6 Gy)	Fold Change MASM + TBI
NM_130817	Fgf3	fibroblast growth factor 3	2.11	1.55
NM_012603	Myc	myelocytomatosis oncogene	3.41	2.54
NM_031598	Pla2g2a/sPLA2	phospholipase A2, group IIA	5.65	2.12
NM_001008321	Gadd45b	growth arrest and DNA-damage-inducible, β	3.0	1.53
XM_001073032	Map3k10	mitogen activated protein kinase kinase kinase 10	2.13	1.02
XM_344798	RGD1306565/Ask1/Map3k5	similar to apoptosis signal-regulating kinase 1	0.42	0.76
NM_001033963	Prkx/PKA	protein kinase, X-linked	1.58	1.34
NM_019294	Cacna1e	calcium channel, voltage-dependent, R type, alpha 1E subunit	2.14	1.08
NM_053873	Cacna1s/CACN	calcium channel, voltage-dependent, L type, alpha 1S subunit	3.30	1.13
NM_021589	Ntrk1	neurotrophic tyrosine kinase, receptor, type 1	0.29	0.61
NM_022702	Taok2	TAO kinase 2	1.66	1.0
NM_024403	Atf4/CREB	activating transcription factor 4	1.54	1.13
NM_024388	Nr4a1/Nur77	nuclear receptor subfamily 4, group A, member 1	13.56	6.78

## Supplementary Materials

The following are available online at: http://www.mdpi.com/1420-3049/21/5/649/s1. Supplementary File 1 lists 3912 differentially expressed transcripts (probesets) by using one-way analysis of variance (*p* < 0.01). Supplementary File 2 lists 2920 differentially expressed probes (FC ≥ 1.5, *p* < 0.05) were selected by comparison between radiation and wild-type (control) samples. Supplementary File 3 lists 765 genes for which radiation-induced alteration of expression were abolished or attenuated by MASM.
